# Case Report: Small Bowel Obstruction Owing to Self-Anchoring Barbed Suture Device After TAPP Repair

**DOI:** 10.3389/fsurg.2021.646091

**Published:** 2021-02-11

**Authors:** Longbo Zheng, Xiangyi Yin, Huasheng Liu, Shouguang Wang, Jilin Hu

**Affiliations:** Department of Gastroenterology Surgery, Affiliated Hospital of Qingdao University, Qingdao, China

**Keywords:** inguinal hernia, TAPP, small bowel, obstruction, postoperative

## Abstract

Groin hernioplasty is the most performed intervention in the adults worldwide, the minimally invasive inguinal hernia repair techniques widely used by surgeons today are transabdominal preperitoneal patch plasty (TAPP) and total extraperitoneal patch plasty (TEP). We report a 62-year-old man with bowel obstruction caused by the use of self-anchoring barbed suture to close the peritoneum 3 days after TAPP. Surgeons using the barbed suture should be alert to this possibility when encountering this complication of intestinal obstruction, so as to avoid more serious consequences.

## Introduction

Since the 1990s, laparoscopic inguinal hernioplasty has been performed more frequently worldwide. The laparoscopic approach can be either transperitoneal or retroperitoneal. Transabdominal preperitoneal (TAPP) repair is commonly performed because of its advantages as a minimally invasive approach, resulting in less postoperative discomfort, better cosmesis, and a shorter hospital stay (1). TAPP inguinal hernia repair involves dissection of the preperitoneal space, mesh repair of the hernia defect, and closure of the peritoneum (2). A barbed suture is often applied to facilitate closure of the peritoneum. One of these novel sutures is V-Loc™ (Covidien, Mansfield, MA, USA), which consists of a unidirectional barbed absorbable thread equipped with a surgical needle at one end and a loop at the opposite end to secure the suture. However, as demonstrated in several case reports (3, 4), a potential drawback to the use of barbed suture is that exposed suture barbs may catch on the adjacent small bowel, mesentery, or omentum and cause serosal injury, obstruction, or volvulus. We herein present a case of postoperative small bowel obstruction (SBO) after TAPP repair that was directly related to the use of barbed suture.

## Case Report

**Figure 1:**
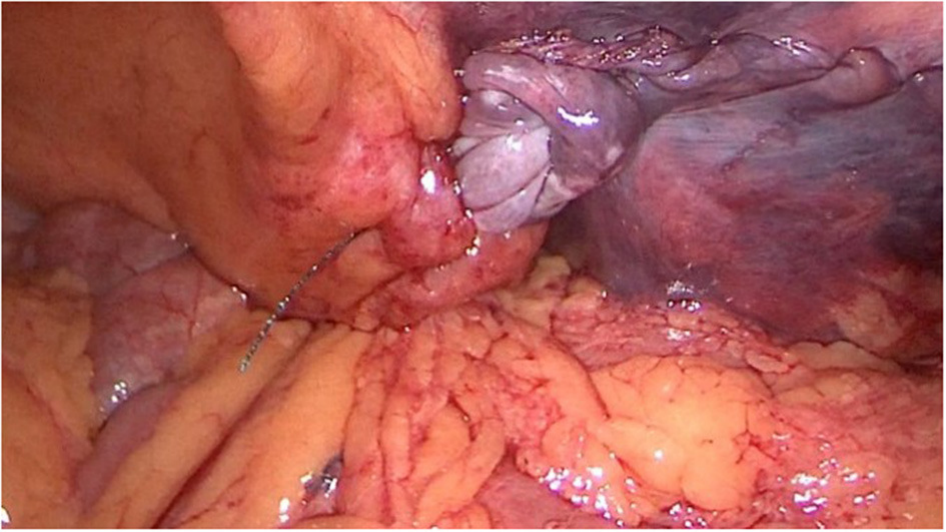
Barbed suture remaining in the abdominal cavity after the initial operation.

A 62-year-old man with a body mass index of 18.5 kg/m^2^ presented with symptomatic bilateral inguinal hernias. The bilateral inguinal hernias were evident on clinical and ultrasound examinations. TAPP repair was performed by an experienced surgeon at the Department of Gastroenterology Surgery, Affiliated Hospital of Qingdao University. After stripping the right hernia sac, a 15- ×10-cm partially absorbable mesh (Ultrapro UMM1, Johnson & Johnson Medical, USA) was fixed at multiple points with 2 mL of medical glue (Compont® Beijing Compont Medical Devices Co., Ltd., Beijing, China) using a laparoscopic sprayer. V-loc™ absorbable barbed suture was used to close the peritoneum, leaving about 2 cm of exposed suture in the abdominal cavity (Figure 1). The patient was in stable condition after surgery and was discharged on postoperative day 1.

**Figure 2:**
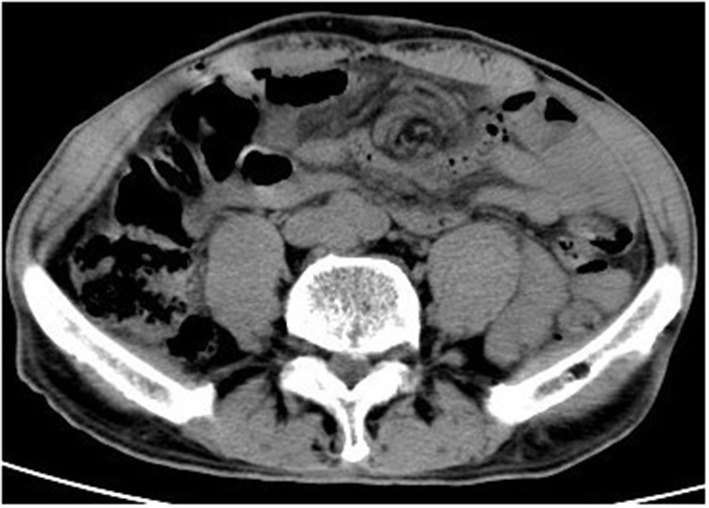
CT of the abdomen on re-admission, the red arrow indicates the “vortex sign” that marks volvulus.

**Figure 3:**
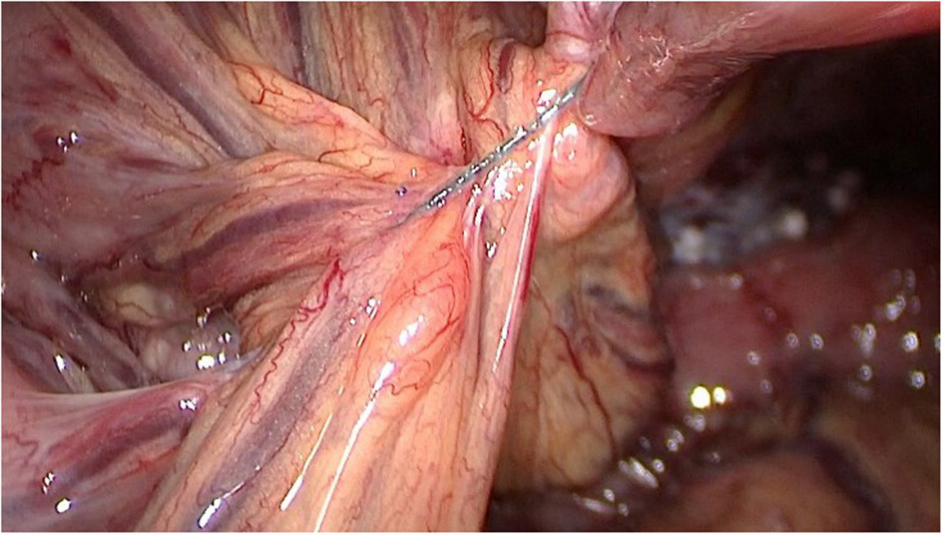
Emergency laparoscopy revealed that the barbed sutures were firmly entangled with the mesenteric.

The patient presented to the emergency room on postoperative day 2 with severe abdominal pain and no flatulence or defecation for 2 days. Abdominal computed tomography showed mild small intestinal dilation and the “whirlpool sign,” consistent with intestinal volvulus (Figure 2). Considering these imaging findings combined with clinical signs consistent with intestinal volvulus, emergency laparoscopy, was performed. The small intestinal membrane was found to be stuck in the lower right quadrant by the reverse hook of the V-loc™ suture that had previously been used to close the peritoneum, and the surrounding intestines exhibited significant congestive edema (Figure 3). After cutting the V-loc™ suture and performing derotation, good reperfusion was obtained. The patient recovered well and was discharged on postoperative day 2. Upon hospital discharge, physical examination revealed no abdominal softness or pressure pain, and he was able to tolerate food and perform bowel movements.

## Discussion

The overall complication rate of TAPP repair is low because of technological advances. Statistical data indicate that the incidence of SBO after TAPP repair ranges from 0.2% to 0.5% (5) and is usually attributed to inadequate peritoneal closure, trocar site herniation, or adhesion (6). We identified other case reports similar to ours, in which a self-anchoring barbed suture remained after TAPP repair and caused SBO (7, 8). Although such cases are rare, they provide many clinical learning points.

Complete closure of the peritoneum during TAPP repair is a very important step. On one hand, it prevents intestinal adhesion and obstruction caused by mesh exposure. On the other hand, it can prevent the intestine from entering the preperitoneal space (2, 9). Closure can be achieved with staples, tacks, running suture, or glue. Among these options, application of running suture is more time-consuming to perform but less painful to the patient (9). In recent years, self-anchoring barbed suture such as V-loc™ has eliminated the need for knot tying and has thus been welcomed by clinicians (10). The efficacy and suitability of barbed sutures have been reported in the fields of gynecologic surgery (11, 12), plastic surgery (13), urology, orthopedic surgery (14, 15), and general surgery (16).

The use of self-anchoring barbed suture saves time but is also associated with several risks. In the present case, the free suture in the abdominal cavity was firmly fixed to the mesentery of the small bowel, forming a rivet point. The mesentery and small intestine became entangled at this point during peristalsis, causing intestinal volvulus. If surgery is not performed in a timely manner in such cases, more serious ischemic intestinal obstruction will occur and intestinal necrosis may even develop. Sartori et al. (17) reported a case of SBO after TAPP hernia repair using a barbed suture. The patient returned 3 days postoperatively with typical signs of bowel obstruction. Additionally, Lee and Wong (18) reported a case of SBO caused by barbed sutures 6 weeks after laparoscopic myomectomy. Api et al. (19) established a rat model and found that barbed suture material might be associated with adhesion formation when used intra-abdominally and that these adhesions could not be prevented by peritonization. Notably, these clinical cases and animal models had a common feature: the barbed suture was left in the abdominal cavity and directly contacted the intestine or mesentery.

## Conclusions

Self-anchoring barbed suture has a wide range of indications and good performance. More importantly, however, it must be used correctly to reduce the risk of complications. The cases discussed herein emphasize that although barbed sutures can be applied more rapidly and conveniently under laparoscopy, the free end of the barbed suture remaining in the abdominal cavity may adhere to the intestine or mesentery, resulting in an intestinal obstruction. This complication can be avoided by either preventing exposure of excess suture in the abdominal cavity or using conventional absorbable sutures for peritoneal closure. A high degree of suspicion for this complication is warranted in patients who show typical signs after surgery. Early diagnosis and treatment should be ensured to avoid adverse consequences such as intestinal necrosis, which will seriously affect the prognosis.

## Data Availability Statement

The original contributions presented in the study are included in the article/supplementary material, further inquiries can be directed to the corresponding author/s.

## Ethics Statement

The studies involving human participants were reviewed and approved by Ethics Committee of the Affiliated Hospital of Qingdao University School of Medicine. The patients/participants provided their written informed consent to participate in this study. Written informed consent was obtained from the individual(s) for the publication of any potentially identifiable images or data included in this article.

## Author Contributions

LZ and XY draft the manuscript. SW, HL, and JH edited the manuscript. All authors were involved in the clinical care of the patient and approved the final version of the manuscript at time of submission.

## Conflict of Interest

The authors declare that the research was conducted in the absence of any commercial or financial relationships that could be construed as a potential conflict of interest.
